# Intraoperative intravital microscopy permits the study of human tumour vessels

**DOI:** 10.1038/ncomms10684

**Published:** 2016-02-17

**Authors:** Daniel T. Fisher, Jason B. Muhitch, Minhyung Kim, Kurt C. Doyen, Paul N. Bogner, Sharon S. Evans, Joseph J. Skitzki

**Affiliations:** 1 Department of Immunology, Roswell Park Cancer Institute, Buffalo, New York 14263, USA; 2 Department of Urology, Roswell Park Cancer Institute, Buffalo, New York 14263, USA; 3 Department of Surgical Oncology, Roswell Park Cancer Institute, Buffalo, New York 14263, USA; 4 Spectra Services, Incorporated, Ontario, New York 14519, USA; 5 Department of Pathology, Roswell Park Cancer Institute, Buffalo, New York 14263, USA

## Abstract

Tumour vessels have been studied extensively as they are critical sites for drug delivery, anti-angiogenic therapies and immunotherapy. As a preclinical tool, intravital microscopy (IVM) allows for *in vivo* real-time direct observation of vessels at the cellular level. However, to date there are no reports of intravital high-resolution imaging of human tumours in the clinical setting. Here we report the feasibility of IVM examinations of human malignant disease with an emphasis on tumour vasculature as the major site of tumour-host interactions. Consistent with preclinical observations, we show that patient tumour vessels are disorganized, tortuous and ∼50% do not support blood flow. Human tumour vessel diameters are larger than predicted from immunohistochemistry or preclinical IVM, and thereby have lower wall shear stress, which influences delivery of drugs and cellular immunotherapies. Thus, real-time clinical imaging of living human tumours is feasible and allows for detection of characteristics within the tumour microenvironment.

## Abstract (web summary)

Intravital microscopy has been used in laboratory animals to visualise the blood vessels in tumours. Here, the authors use this technique in melanoma patients undergoing surgery and show that vessels *in situ* have a larger diameter than excised tissue

## Graphical abstract

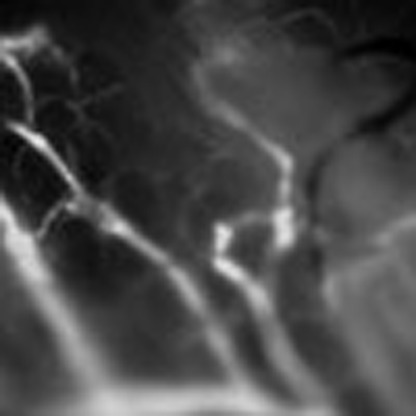

Recent advances in the preclinical imaging of tumours have generated an unprecedented understanding of the nature of tumour vascular structure, blood flow and tumour-immune cell interactions. As the gateway to the tumour microenvironment, tumour vessels are critically important sites that serve to enhance or limit host interactions. Immunohistochemistry (IHC) and pathologic examination remain the clinical standard for defining tumour characteristics including vascularity, but provide only a static ‘snapshot' that may not reflect the active processes occurring *in situ* in a living, growing tumour. Common clinical imaging procedures such as retinography (≤ × 15 magnification) have proved useful for examination of vessels at the macrolevel. However, preclinical intravital microscopy (IVM) at typical magnifications of ≥ × 100 magnification is fundamentally more powerful for the real-time microscopic evaluation of the dynamic nature of functional blood vessels within living organisms at a resolution capable of distinguishing cellular and subcellular features within the surrounding microenvironment^1^.

As a research tool, preclinical IVM in murine systems has contributed to the elucidation of inherent tumour functions including angiogenesis^1^,^2^,^3^,^4^, metabolism^5^,^6^, metastasis^7^,^8^ and immune cell interaction^9^,^10^,^11^,^12^. Observations on a microscopic level have allowed the tumour vasculature to be measured for parameters such as vessel diameter, blood velocity, ‘leakiness' of the endothelium and potential vessel ‘normalization' responses to targeted vascular therapies^1^,^12^. These critical vascular parameters serve as the foundation for calculations associated with tumour blood flow, wall shear stress and perfusion, which directly impacts tumour metabolism, oxygenation, angiogenesis, drug delivery and immune cell interactions. Direct observation of tumours by IVM in rodents has allowed insight into many critical facets of tumour biology including permissiveness to chemotherapy agents, metabolism, hypoxia and acidosis^2^,^13^,^14^,^15^,^16^. Furthermore, tumour-immune cell interaction together with lymphocyte trafficking within the tumour microenvironment are increasingly recognized determinants of cancer outcome^9^,^12^,^17^ and can be interrogated by IVM. Clinically, quantification of all of the aforementioned variables via real-time IVM of human tumours could be directly applied not only to prognosis, but also eventual treatment decisions based on direct observations of the tumour microenvironment. Although IVM studies evaluating biology in murine tumour models have spanned >70 years^18^, to this point there has not been a single report using this technology to evaluate human malignant disease. Thus it remains unclear whether the significant discoveries using IVM in small animal models can be reliably extrapolated to humans.

Barriers to performing IVM in humans have been numerous. Successful measurements of vessel diameter, density, blood velocity and wall shear stress by IVM requires visual access to the tissue of interest, the ability to detect contrast agents administered to the organism, and *post hoc* movie analyses of recorded observations. Preclinical animal models often utilize window chambers that facilitate visual access to underlying tissue during IVM (refs 13, 15), but these implanted devices are not feasible in humans. The dearth of clinically available and optimized epifluorescent probes has also been a major obstacle to performing IVM in humans. We hypothesized that the technical barriers to IVM of human cancer could be overcome, allowing an unparalleled view of dynamic tumour processes. Preclinical studies by our group and others include the routine observation of mouse lymph node vasculature without the use of window chambers^11^,^19^,^20^,^21^,^22^. We reasoned that superficial human primary and nodal metastatic melanoma tumours, which have been extensively studied in regards to metastasis, metabolism and immune cell interaction, would be similarly accessible for direct visualization at the time of surgical resection, thus, obviating the need for window chambers. Surveying the limited list of Food and Drug Administration (FDA)-approved non-toxic, fluorescent substances^23^, the venerable drug fluorescein appeared to be the most applicable as a natural extension of our preclinical IVM studies^9^,^12^,^22^ and is commonly used during surgical procedures to detect tissue perfusion on a macro level^24^,^25^,^26^, thereby providing a precedent for the study of human tumours by IVM.

Herein, we report on the application of a newly developed intraoperative epifluorescent portable platform combined with existing surgical techniques. The clinical application of this technology is feasible with a focus on vascular parameters that have a direct relationship to host interactions including tumour vessel diameter, density, blood velocity and wall shear stress. IVM of human tumours confirms the findings of tumour vessel disorganization, tortuosity and limited functionality as commonly seen in preclinical models^1^,^4^,^6^. However, unexpected and significant differences are present regarding tumour vessel diameters measured by IVM as compared with IHC or preclinical IVM which translated to substantial lowering of calculated wall shear stress. The unique, real-time measurement of human tumour vessel diameters *in vivo* has implications for future investigation of tumour metabolism, oxygenation, chemotherapy delivery, anti-angiogenic therapy and immunotherapy.

## Results

### Evaluation of patient tumour vessels by IVM

**Figure 1:**
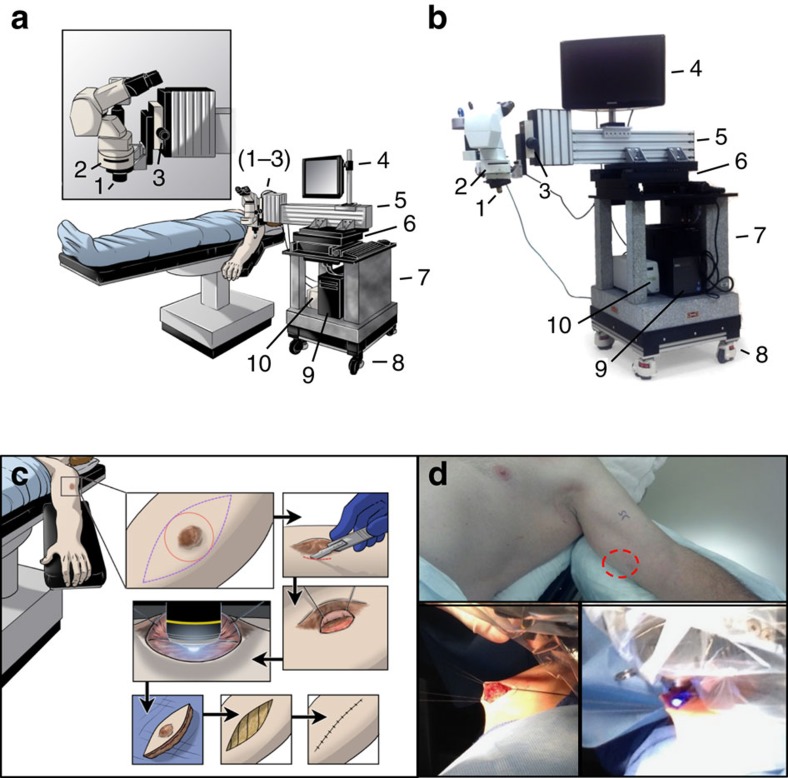
Design of a portable intraoperative IVM system and surgical exposure of tumour microvasculature. (**a**) Schematic and (**b**) photograph of the intravital microscopy unit designed as a mobile system for observations in the OR. Critical systems allowing for stable epifluorescence observation of patient tumours in real-time are highlighted as follows: (1) × 10 objective lens (× 100 total including × 10 subjective lens), (2) fluorescent filter cube, (3) fine focus knob, (4) monitor to observe captured movie in real time, (5) cantilevered arm to extend the system over the patient, (6) heavy movement platform to control fine *x*–*y* motion of the system during observation, (7) solid granite base to reduce vibrations, (8) locking wheels, allowing mobility and stability, (9) integrated computer system to record observations and store data and (10) fluorescent light source. (**c**) Schematic and (**d**) representative photographs, detailing the surgical procedure to allow for observation of tumour microvasculature. Briefly, an incision is made in the skin overlying or adjacent to the tumour (red circle) to allow access for direct tumour observation. The epifluorescent light source is activated and digital movie recording commenced to observe the intravenous injection of fluorescein. Following observation the access site is closed and a standard oncologic resection performed.

Herein, we report the results of the first arm of a clinical trial that determined feasibility in 10 patients who underwent intraoperative IVM. The microscopic platform was engineered with a focus on mobility in the operating room (OR) and subsequent vibration-limiting stability when interfaced with the tumour. To create a working space between the microscope objective and the patient positioned on the OR table, a simple cantilever construction was chosen (Fig. 1a,b). The cantilever design was anchored to a granite base weighing >360 kg with the ability to move the apparatus into position and then ‘lock' to the OR floor in a fixed position, thus providing a high level of stability. The light source, computer, and monitor required for digital microscopic movie acquisition were integrated into the granite design to streamline mobility of the entire platform.

Melanoma patients enrolled in our Roswell Park Cancer Institute review board-approved protocol (ClinicalTrials.gov identifier: NCT01886235) met strict eligibility criteria that included having a melanoma that was either palpably or visibly ≥0.5 cm that required excision in the OR as their standard of care (Supplementary Table 1). Eligible patients of any stage melanoma that required surgical excision consented to a brief, 15 min IVM observation period during the standard course of their planned surgery at Roswell Park Cancer Institute. Patients were pre-tested for sensitivity to fluorescein with a skin-prick test with a positive reaction indicating an increased risk of anaphylaxis^27^. All patients tested negative for fluorescein sensitivity and proceeded to IVM observation in the OR.

During surgery, microscopic observations were performed first, before the definitive oncologic surgical procedure to eliminate any potential artifact. In some superficial melanomas that attenuated the overlying skin, direct IVM observations were performed without any manipulation of the tumour. For the observation of deeper lesions, the incision for the microscopic observation was delineated in a manner consistent with standard oncologic resection, yet also allowed for a ‘portal' to observe the tumour surface directly without disrupting tumour-feeding blood vessels (Fig. 1c,d). The platform was moved into position and mechanized controls connected to the base were used to align the exposed, and often pigmented, tumour tissue with the microscopic objective before IVM observation and digital recording. Body mass index and tumour depth did not influence the ability of the portable microscope to interface with the tumour tissue and image stability was sufficient to allow accurate measurements with a spatial resolution in the range of 1 μm as determined by micrometre measurements. An IVM observation was considered successful if the tumour vessels could be identified, vascular diameter and density measured, and intravascular fluorescein observed in real-time. Despite the varied patient characteristics and tumour location, the intravital microscope was successful in the majority of patients at different anatomic locations and tissue depths (Supplementary Table 2).

**Figure 2:**
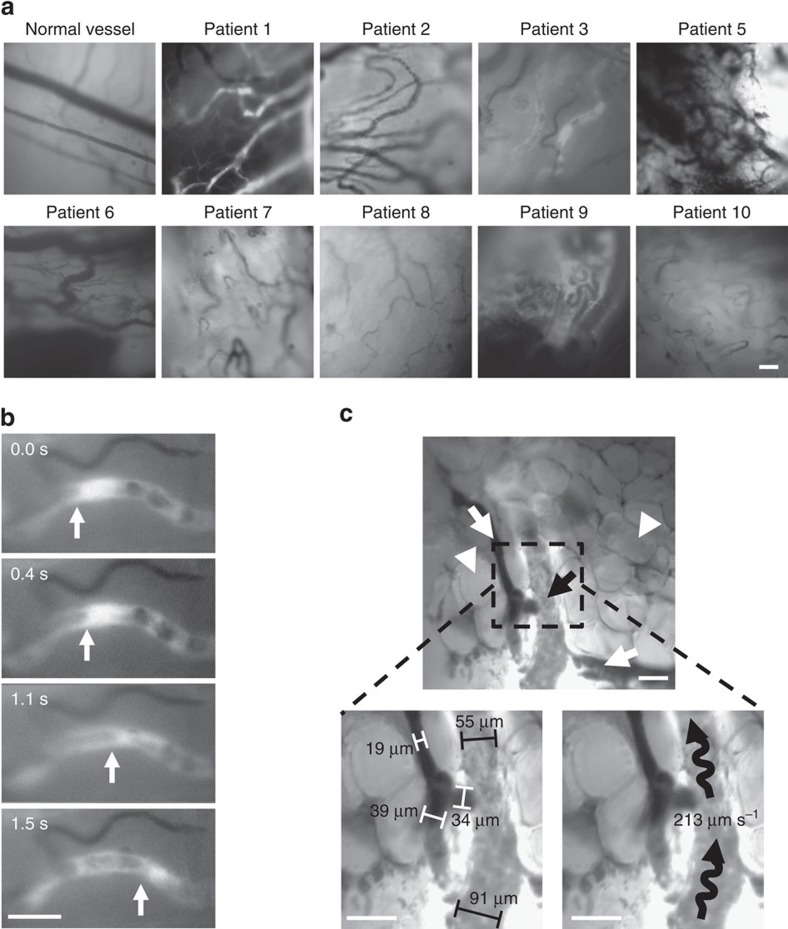
Detection of blood flow in patient tumour microvasculature. (**a**) Representative photomicrographs of tumour microvasculature in patients following fluorescein injection. (**b**,**c**) Photomicrographs exported during movie analysis of blood flow velocity. (**b**) Arrow denotes the trailing edge of a bright fluorescein signal that was utilized to determine blood flow velocity in mm s^−1^. (**c**) Measurement of tumour diameters and blood vessel flow rate. White arrows indicate vessels that did not support blood flow during observation, black arrows denote functional vessels and white arrowheads show adipocytes. Brackets depict tumour diameter measurements of non-functional (white) and functional (black) vessels with wavy black lines demonstrating direction of blood flow and flow rate. (**a**–**c**) Bar is 100 μm.

Measurements of vessel density and diameter were possible in some patients even before the injection of fluorescein due to the absorptive capacity of haemoglobin in red blood cells. However, due to the dense melanin pigmentation of the melanoma tumours visualization of the vessels was greatly improved following injection of fluorescein. Typical images of patient melanoma tumours included evidence of surrounding adipose tissue and vessels of varying depths within the tumour tissue (Fig. 2a). Tumour vessels were noted by their tortuosity and convoluted architecture, which was in stark contrast to normal linear vessels detected in the surrounding skin (Fig. 2a). Fluorescein extravasated from the vessels into the tumour tissue immediately and was marked by an intense fluorescent signal. This increase in fluorescence required observations to be dynamically adjusted to compensate for ‘burn in' by the acquiring software (Supplementary Movie 1). *Post hoc* application of image stabilization software (for example, ImageJ) did not appreciably change the degree of motion artifact.

### A high proportion of tumour vessels do not support blood flow

During the course of observation, a variable percentage of vessels within the tumour appeared patent, but did not manifest any evidence of blood flow or intravascular fluorescence following fluorescein injection (Table 1). For the purposes of analysis, vessels that lacked perceptible blood flow or intravascular fluorescence for the duration of observation were considered ‘non-functional'. In evaluable patients, on average, 49±7% (Mean±s.e.m. *n*=7) of vessels were deemed non-functional at the time of observation despite most patients having a mean arterial pressure exceeding 65, which is considered adequate for tissue perfusion^28^. To exclude the possibility that non-functional vessels were related to surgical exposure or tissue trauma, transcutaneous microscopic observations were performed in Patient #2, #7 and #10 before any surgical manipulation. These patients had large nodular melanomas that attenuated the overlying skin and tumour vessels were clearly visible transcutaneously which offered a unique opportunity for examination by IVM without tissue perturbation (Supplementary Fig. 1a–c, Supplementary Movies 2,3). The transcutaneous observations demonstrated an average of 53±6% (Mean±s.e.m. of the mean *n*=7) of tumour vessels that were non-functional, further suggesting that this is an inherent characteristic of the undisturbed tumour.

In three patients (#4, #5 and #9) intravascular fluorescein could not be detected in the exposed tumour, and thus, blood flow velocity and per cent of non-functional vessels could not be calculated. Patient #4 had a profound desmoplastic reaction around his tumour, which likely was a physical barrier preventing visualization of underlying tumour vessels. Subsequent histology of this tumour confirmed a vigorous fibrotic reaction (Supplementary Fig. 2). In Patients #5 and #9, fluorescein could not be detected within the vascular lumen, but non-contrasted vessels were readily identifiable and measured for density and diameter.

### Vessel diameters and shear stress are larger than predicted

Post-movie acquisition analyses over multiple areas and vessel segments provided extensive data regarding tumour vessel characteristics. The digital recordings of each field of observation were analysed for tumour vessel density, diameter and blood flow velocity for each tumour (Table 1). Remarkably consistent measurements were noted with a mean of 8±1 vessels per field and an average diameter of 31±3 μm measured during IVM. The larger vessel diameters of patient tumours measured by IVM were in stark contrast to typically reported smaller values derived from human tumour IHC or preclinical animal models (for example, 15 μm)^29^,^30^. Blood flow velocity was measured in the vessels based on visual tracking of the movement of discrete fluorescence signals caused by heterogeneity in the fluorescein dye and averaged 286±23 mm s^−1^ (Table 1, Fig. 2b,c, Supplementary Movie 4).

**Figure 3:**
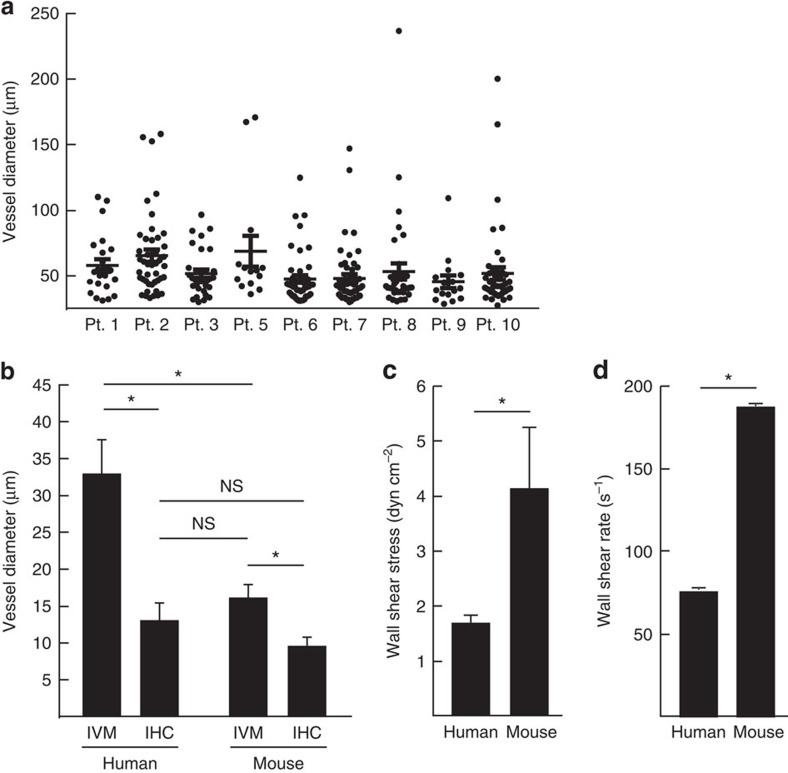
Comparison of tumour vessel diameters by different methodologies. (**a**) Individual measurements of tumour vessel diameters in patients. (**b**) Tumour vessel diameters quantified by human IVM and IHC of the same tumour tissue and murine melanoma tumours demonstrate consistently larger diameters detected by IVM. Established methods of estimating human tumour vessel diameters by IHC or mouse IVM produce similar measurements that both significantly underestimate diameters measured by human IVM. (**c**) The wall shear stress in tumour vessels is larger in murine tumours when compared with patient tumours. (**d**) Wall shear rate is lower in patient tumour in comparison to patient tumours. (**a**–**d**) *P<0.05l; NS, non significant (unpaired Student's *t*-test). *n*=9 for patients; *n*=10 for mice. Data is mean±s.e.m.

To determine how these intraoperative IVM findings compared with standard pathologic evaluation, tumour specimens from the same patients were stained for CD31 to calculate vessel density and diameter (Table 1). IHC is the gold standard for pathologic evaluation of tumour tissue but can only offer a static, one-time view of the tumour vasculature. As such, a comparison of dynamic data such as blood flow velocity, vessel patency and function as measured by IVM could not be performed. Strikingly however, while the vessel diameters measured by IHC averaged 14±1 μm and were in line with typically reported values^29^, these measurements were consistently and significantly smaller than those recorded by IVM (31±3 μm) of the same tumour tissue yielding a mean difference of 17 μm (Table 1, Fig. 3a,b).

The tumour vessels diameters recorded by IVM in patients tumours were also substantially larger than established estimates from previously reported values of preclinical animal models (15 μm)^30^ and those obtained by our group in murine tumours implanted in dorsal skin-flap window chambers (that is, B16 13.2±1 μm, Supplementary Table 3)^12^. Therefore, we sought to determine how the vessel diameter measurements in patient tumours by IVM compared with preclinical models using the same microscope platform and surgical procedure. Established B16 murine melanoma tumours implanted subcutaneously in the distal thigh of C57BL/6 mice were interrogated using IVM, without a window chamber, in a manner identical to this clinical trial, thus eliminating potential variability between the clinical and preclinical setting as well as any differences in resolution or technique (Supplementary Fig. 3). Using this IVM platform, mouse melanoma tumour vessel diameters measured on average 15±2 μm (Fig. 3b). The increased diameter of patient tumour vessels versus preclinical models also had dramatic effects on calculation of vascular parameters such as wall shear stress and wall shear rate, which are critical factors mediating the ability of lymphocytes to gain access to tissues sites from the blood^31^,^32^,^33^. The larger diameter of the vessels combined with the unchanged blood flow velocity further yielded a lower wall shear stress (1.7±0.1 dyn cm^−2^) and wall shear rate (77±5.6 s^−1^) in human tumours than would have been predicted based on studies in B16 melanoma murine tumours shown here (4.1±1.1 dyn cm^−2^ and 188±50 s^−1^ for wall shear stress and wall shear rate, respectively) (Fig. 3c,d, Supplementary Table 3) and in our prior reports^12^. To ensure that these differences between human and murine vascular parameters were not a function of the B16 melanoma tumour model, several other murine tumours were examined by IVM including CT26 colorectal carcinoma, EMT6 mammary carcinoma and 4T-1 mammary carcinoma. All murine models examined showed statistically smaller tumour vessel diameter measurements and higher wall shear stress when compared with patient tumour vessels (Supplementary Table 3) suggesting that these differences were independent of the murine model chosen. To determine if the difference in haemodynamic properties between human and mouse melanoma tumour vessels was a product of general anaesthetics, which have known vasodilatory properties^34^, that could impart larger tumour vessel diameter measurements, mice received the same class of general anaesthetic used in our patients (isoflurane). No difference in tumour vessel diameters was noted during IVM whether the vasodilatory anaesthetic isoflurane or a ketamine/xylazine combination that maintains vascular tone was employed (Supplementary Fig. 4).

Another potential explanation for the differences in human tumour vessel diameters measured by IVM versus IHC could be related to tissue shrinkage following formalin fixation. To address this possibility, we examined how vessel diameters were altered in the B16 murine model by taking the IVM observed murine tumours and examining the vessel diameters by IHC. The murine tumours were treated in an identical fashion to the patient tumours including the same fixation time, paraffin embedding process, and tissue sectioning and IHC staining. The average diameter of murine tumour vessels measured by IHC was 9±1 μm and demonstrated a significant reduction in diameter as compared with the diameters detected by IVM in mice (15±2 μm), which suggested process-related tissue shrinkage (Fig. 3b). However, as the disparity noted between murine IVM and IHC was much less than between human IVM and IHC, formalin fixation was unlikely the sole reason for the significantly larger vessel diameter measurements detected by IVM in patient tumours.

### Vessel diameters are similar between periphery and core

**Figure 4:**
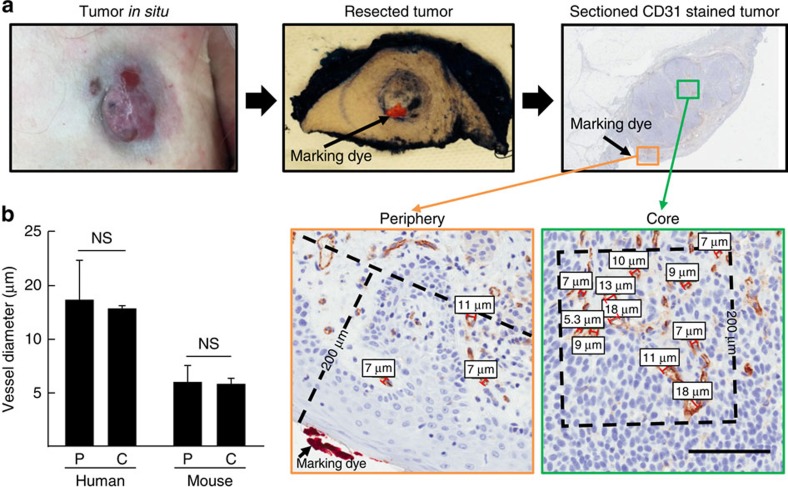
Vessel diameters are similar in the periphery and core of tumours. (**a**) Marking dye was applied following surgical resection to identify the imaged surface of tumour. Measurements of core (>200 μm from surface) and peripheral (<200 μm from surface) tumour blood vessels was performed on formalin-fixed tumour samples stained using haematoxylin and anti-CD31 mAb. Bar is 100 μm. (**b**) Comparison of tumour vessel diameters per field (minimum 5 fields) in the periphery (P) versus core (C) in humans and mice, NS, non significant (unpaired Student's *t*-test). *n*=3 for patients; *n*=5 for mice. Data is mean±s.e.m.

A potential explanation for the disparity between tumour vessel diameters measured by IVM and IHC could have been that the interstitial pressure in tumours is variable between the core and the periphery^35^ and epifluorescence IVM observations are limited to the tumour surface. Thus, tumour vessel diameters were examined by transcutaneous IVM in the periphery and IHC in both the periphery and the core to determine if the discrepancy in tumour vessel diameters may have been a result of a greater sampling of vessels towards the core of the tumour. This allowed for the periphery and core of theses tumours to be readily defined and in the case of Patient #10, the site of IVM-tumour interface was marked with ink at the time of surgical resection (Fig. 4a) so that tumour vessels in the same regions could be measured by both IVM and IHC for direct comparison. No significant differences were noted in diameters of vessels measured in the periphery as compared with the core of the tumour (Fig. 4b). These findings reduce the possibility that the larger diameters of tumour vessels detected by IVM are due to a sampling bias of peripheral vessels. Complimentary studies performed in mouse melanoma tumours demonstrated that vessel diameters examined by IHC in the periphery versus the core showed no significant difference, confirming our observations in human tumours (Fig. 4b, Supplementary Fig. 5).

## Discussion

Current innovations have made human IVM possible and allowed the transition of this technology from the laboratory to the clinical study of cancer. In the current feasibility trial, we have demonstrated the ability of IVM to successfully measure dynamic tumour vascular features for the first time in humans with a spatial resolution of 1 μm. The method of IVM observation of tumours was seamlessly incorporated during surgical resection and did not interfere with the standard operative procedures. Our platform was capable of accessing any anatomic location on a patient and digital movie recordings demonstrated high resolution and manageable motion artifact that allowed for precise measurements of tumour-associated vessels. The results demonstrate that live-imaging of human tumour dynamics at a microscopic level (≥ × 100 magnification) is feasible, consistent and reproducible.

Intravital imaging in melanoma patients confirmed expectations for several key vascular parameters including the presence of tortuous, disorganized architecture and non-functional tumour vessels^36^. Importantly, tumour vessels that did not display fluorescein uptake were present even without any surgical manipulation of the tumour as evidenced by the transcutaneous observations. Since IVM is inherently limited by the depth of epifluorescent light penetration (that is, maximum of 200 μm)^37^, it cannot be determined whether the percentage of functional vessels vary within deeper tumour regions. However, it has been well established that tumour vasculature is heterogeneous^1^,^4^,^38^, thus we attempted to interrogate as many possible areas of the exposed tumour to generate a broad view that was representative of the entire lesion. As the IVM generated measurements were fairly consistent both within individual tumours and between patients, this would suggest an adequate sampling of tumour fields and measurement accuracy. Despite a limited view of deeper tumour regions, the collection of data regarding the tumour vascular blood flow and architecture from superficial vessels may offer clinically relevant prognostic information as has been previously described in animal models^39^,^40^ and preliminary human studies^41^,^42^. For example, the detection of microvascular loops and networks by standard IHC in ocular melanoma vessels has been directly correlated to decreased survival of patients^43^,^44^. Therefore, our IHC analysis of tumour vasculature suggests that data acquired by IVM observation of superficial tumour vessels may be extrapolated to deeper vessels beyond the reach of epifluorescence microscopy. It is possible that IVM of human tumours may reveal vessel patterns and characteristics that can be eventually linked to tumour aggressiveness and patient prognosis.

The major finding from this clinical trial was the consistent and significantly larger tumour vessel diameter measurements obtained by IVM versus IHC in patient tumours. As confirmation that the differing measurements were not due to sampling bias, IHC measurements were performed in the same fields observed during transcutaneous IVM. Tumour vessel diameter measurements by IHC remained significantly smaller despite matching the fields observed by IVM. Also, the ‘shrinkage' of tissue that typically occurs with formalin-fixation^45^,^46^ is only partially responsible when comparing the degree of tissue shrinkage in humans and mice. Most likely the larger lumenal diameters seen by IVM in human tumours were due to the natural distention of functional, dynamic vessels as a result of mean arterial pressure during the observation of living tissue. Since there was good uniformity in IHC tumour vessel diameter measurements between the periphery and core of the melanoma tumours, it is unlikely that the larger tumour vessel diameters seen by IVM were related to a biased sampling of potentially larger superficial tumour vessels. These results resemble findings from prior studies utilizing corrosion casting of human tumour microvasculature in regards to tumour vessel tortuosity and disorganized architecture^47^, and similar to IHC, this casting technique also underestimated peripheral tumour vessel diameters (19.4 μm) compared with human IVM (33 μm). Interestingly, the tumour vessel diameters measured by IVM in a preclinical mouse model of melanoma using the same exact methodology and microscope also significantly undervalued the diameters seen in IVM of human melanoma tumours. Regarding observed differences between human and mouse IVM, the diameters of tumour vessels detected by IVM in preclinical models may be species-specific and potentially related to the nature of rapidly growing, implantable murine tumours that differ from the protracted growth trajectory of orthotopic tumours found in humans. Overall, the disparate vessel diameters measured between IVM and IHC in patients and measurements in murine models indicate that extrapolations regarding human tumour vessel structure from IHC or preclinical IVM may not accurately represent the hemodynamic parameters within patient tumour vessels.

The finding of consistently larger tumour vessels by human IVM could be profound when considering how the geometry of blood vessels influences tumour tissue perfusion and drug delivery as well as trafficking of immune cells into the tumour site. For example, a recent review on tumour vessel normalization cited a standard tumour vessel diameter of 15 μm (ref. 30), which is in direct line with dimensions in the literature and those observed in the current study using IHC of patient tumours and IVM in mice, but substantially undervalues the consistently larger diameters that are measured by human IVM (average 31±3 μm). The consequences of inaccurate diameter measurements are magnified when considering that this vascular parameter is critical for calculating vessel wall shear stress^31^,^32^,^33^. Wall shear stress is a necessary component in many biological processes controlling shapes of various cell types within the blood compartment (for example, red blood cells, leucocytes and endothelial cells), gene transcription, kinase activity, nitrous oxide production, vascular permeability and lymphocyte trafficking^31^,^32^,^33^,^48^. High wall shear stress has been linked to decreased permeability in porcine arterial endothelium^48^, so the lower than expected wall shear stress is in line with the immediate detection of fluorescein extravasation observed in the current study. Although the permeability of the vessels was not directly addressed in the current study, fluorescein, with a molecular weight of 332 g mol^−1^, could be considered as a surrogate for common chemotherapeutic agents with a similar size such as 5-FU (130 g mol^−1^), cyclophosphamide (261 g mol^−1^) and oxaliplatin (397 g mol^−1^). Moreover, certain chemotherapy agents, including doxorubicin, are auto-fluorescent^49^ suggesting that detection of drug distribution in real-time by IVM in patients is possible.

Numerous *in vitro* and *in vivo* studies have further documented that mechanical forces associated with vessel diameter and wall shear stress have a profound impact on lymphocyte trafficking. In this regard, a narrow window of wall shear stress ranging from ∼0.5 to <2 dyn cm^−2^ is necessary for optimal lymphocyte adhesion to vessel walls during trafficking, whereas adhesion within vessels declines precipitously when the shear stress exceeds 3 dyn cm^−2^ (refs 31, 32, 33). Thus, the wall shear stress described here and in our prior report^12^ that consistently exceeds 3 dyn cm^−2^ for B16 and other murine tumours would suggest that the hemodynamic forces are a substantial hurdle to the trafficking of immune cells across the tumour endothelium. In sharp contrast, the lower wall shear stress of 1.7±0.1 dyn cm^−2^ found in human tumour vessels raises the possibility that the tumour microvasculature could be a permissive site for delivery of T-cell-based immunotherapeutics, which has important implications in the context of adoptive T-cell transfer therapy, immune checkpoint blockade therapy, and dendritic cell vaccines.

In summary, the application of IVM in humans has been demonstrated to be feasible and has begun to produce real-time, functional characterization of tumour vessels as the main interface of tumour-host interactions in their dynamic state. The potential applications of this technology are abundant in the field of clinical oncology and are not limited to melanoma tumours. Melanoma tumours were chosen for this inaugural trial of human IVM as they are typically superficial in the dermal or subcutaneous tissue. While the associated dense pigmentation of melanoma tumours allowed for confirmation of microscopic localization within bona fide tumour tissue, it also made blood vessel visualization before fluorescein injection more difficult. Also, a brisk desmoplastic reaction prevented IVM observation in one melanoma patient which could not have been predicted before surgery. The exposure of the melanoma tumours required a delicate skin-flap technique that would be unnecessary for other tumour types within the peritoneal or pleural cavities. It is anticipated that virtually any tumour-type in any anatomic location could be observed using this platform to provide detailed analyses of inherent tumour physiology, drug delivery and immune cell trafficking.

## Methods

### Patient selection

Patients with a visible (>0.5 cm in maximal diameter) or palpable melanoma lesion that required excision in the OR were evaluated for entry into clinical trial I 231512 ‘A Pilot Study of Feasibility of Performing Intravital Microscopy in Melanoma Patients' at Roswell Park Cancer Institute, Buffalo, NY (ClinicalTrials.gov identifier: NCT01886235) approved by the Institute Review Board of Roswell Park Cancer Institute, Buffalo, NY. Inclusion and exclusion criteria are listed in Supplementary Table 1. Eligible patients who gave informed consent were enrolled in the protocol and information was recorded including demographic data (age, gender, height and weight), tumour-specific data (tumour type, location, depth from skin and stage) as well as photographs from the surgery. Although the reported risk of anaphylactic reaction to fluorescein is low (1 in 222,000), a skin-prick test was performed on all entered patients to determine potential sensitivity^27^. At least 24 h before surgery and the planned microscopic observation, the volar aspect of the patient's non-dominant forearm or opposite side of disease was prepped with an alcohol pad. Three separate areas were demarcated for testing with a marking pen and solutions consisting of 10 μl of 10% fluorescein, histamine chloride (10 mg ml^−1^; positive control) and 50% saline solution (negative control) were placed appropriately. The skin underlying each solution was individually pricked with separate and sterile Duotip lancets (Lincoln Medical, USA). After 30 min, the appearance of a wheal >3 mm in diameter was considered a positive test result in the context of the appropriate positive and negative control. If a positive test was noted to fluorescein, the patient was no longer eligible for this study.

### Microscope design

Patient tumours were observed at × 100 magnification using a highly modified Olympus microscopy system. The microscope was attached to a cantilevered arm to extend over the patient. Given the high magnification required to observe individual vessels and flow, vibrations in the microscope were dampened using a weighted marble base of over 360 kg. Fluorescein was illuminated using a 467–498-nm excitation, 513–556 emission dichronic filter set (Spectra Services, Rochester, NY) with an X-Cite 120 Led light source (Lumen Dynamics, Ontario, Canada). Images were captured using a Luca EMCCD camera (Andor Technology Ltd., Belfast, Northern Ireland) controlled through the Solis acquisition and analysis programme. Images were acquired with a minimum of a 0.05-s exposure at 20 frames a second. Offline quantification of vessel diameter and blood velocity was performed using the ImageJ software suite.

### Surgical technique

Before definitive surgical excision, melanoma tumours were exposed in a consistent manner. The skin overlying the tumour was prepped with ChloraPrep (CareFusion Corp. San Diego, California) and sterile towels were applied for exposure of the area. A curvilinear incision within the field of planned resection was created with a scalpel in line with the longitudinal axis of the patient. With care to keep the vasculature intact, the skin was elevated bluntly and retracted laterally with the use of zero silk sutures. Electrocautery was avoided as much as possible to prevent underlying tissue damage. The sutures were anchored to a retractor affixed to the OR table on the opposite side of the planned microscopic observation. For deeper melanoma tumours, a self-retaining weitlaner retractor was used following incision of the skin and underlying subcutaneous tissue. In all instances, a saline-soaked gauze was placed over top of the tissue to prevent desiccation before microscopic interface. After exposure of the underlying tumour mass, the microscope was positioned for observation. At the completion of the observation period, the microscope was withdrawn and the skin flap was closed with a running 3-0 silk suture. The area was then re-prepped for the definitive oncologic surgery including a new skin prep, sterile towels and drapes, and new gown and gloves.

### Intravital microscopic observations in patients

The microscope was grossly moved into position by the surgeon over the field of interest and locked into place. Fine adjustments in the *x* and *y* axis were performed by using the built-in motor controls. Once the microscope was directly overlying the exposed tumour tissue, the fluorescent light source and image acquisition software were activated. Vertical control of the microscope was performed manually by the surgeon until tumour vessels came into view on the digital monitor. Tumour vessels were clearly recognized by their bizarre architecture, coiling, and branching patterns and observations focused on these areas. To facilitate stabilization of the images, respirations were temporarily held by anaesthesia for a maximum of 30 s. When a stable view of the vessels was achieved, 0.5 ml of 25% (250 mg ml^−1^) fluorescein (Akorn Inc., Lake Forest, Illinois) was injected via a peripheral intravenous catheter. An observation was completed when fluorescein was noted to have extravasated into the tumour tissue and the vessels no longer manifested a detectable fluorescent signal. The microscope was then re-positioned with fine motor controls to observe a different tumour area. The microscopic observations were finalized when the 2 ml of fluorescein was exhausted.

### IVM in murine tumours

For intraoperative IVM experiments 10^6^ B16/F10 melanoma cells^12^, established from mycoplasma-free frozen stocks, were injected subcutaneously in the hind leg of 8–10-week-old female C57BL/6 mice (National Cancer Institute). Experimental analysis was performed 2–3 weeks post-implantation. The skin overlying the tumour was incised and retracted with care to keep the vasculature intact. Vessels were visualized and movie recorded by the same epifluorescent intravital microscope platform used in humans. Lumenal cross-sectional diameter (*D*) of vessels was measured in offline observations of five mice. 4T-1 mammary carcinoma cells and B16/F10 melanoma cells were implanted in C57BL/6 mice (National Cancer Institute; 8–10 weeks) and EMT6 mammary carcinoma cells and CT26 colon carcinomas in BALB/c mice (National Cancer Institute; 8–10 weeks) for correlative window chamber studies as reported^12^. Animal protocols were approved by the Roswell Park Cancer Institute Institutional Animal Care and Use Committee, Buffalo, NY.

### *Post hoc* analyses

Vessels were defined morphologically and a single vessel was described as beginning at a branch point continuing to the next branch point. To be measured, vessels had to be 100 μm in length with no branch points. Vessel density was established by enumerating vessels within a field of observation. ImageJ software was used to measure vessel diameter (*D*) and radius (*r*) at the vessel's largest width. Blood flow velocity (*v*) was evaluated by determining the time Δ*T* distinct features in the fluorescent dye would take to travel a known distance Δ*S* and then averaging *ΔS*/Δ*T* for at least 10 points per vessel. Per cent functionality was determined as mean±s.e.m. on a per field basis. Wall shear stress was computed as (*τ*=32*ηQ*/[*πD*^3^], where *η* is blood viscosity (assumed to be 2.2 centipoise^31^)) and *Q* is the blood flow rate ([*Q*=v*πr^2^] with *r* being the radius of the tumour vessel). Wall shear rate was determined using the formula (*w*=v*8/*D*).

### Tissue processing

Histologic sections were stained with standard haematoxylin and eosin and for CD31 (Human—1:50 dilution, Clone JC70A, Dako, Carpinteria, CA, Murine—1:20 dilution, Clone SZ31, Dianova, Hamburg, Germany). Stained sections were scanned with an Aperio Scanscope XT (Leica Microsystems Inc., Buffalo Grove, IL) and evaluated using Aperio Spectrum software. Vessel density was assessed using standard ‘hot spot' methodology^50^. For each tumour, vessels in five separate hot spot fields were counted at × 200 total magnification. In these same fields, the maximum cross-sectional diameter was measured in all vessels with an identifiable lumen. Measurement utilized the Spectrum software toolset. To distinguish vessels in the periphery versus core, a distance of 200 μm was measured from tumour edge to define a boundary and vessels measured in this region consisted of the periphery.

### Statistical analyses

Unpaired Student's *t*-tests were utilized to examine differences between vessel diameters measured by human IVM, IHC and murine IVM, IHC, comparing each set separately using Graph Pad Prism following testing for data normality with the D'Agostino & Pearson omnibus normality test. No statistical method was used to predetermine sample size. Statistical significance for all comparisons was accepted at *P*<0.05. The numbers of samples per group (*n*), or the numbers of experiments, are specified in the figure legends.

## Additional information

**How to cite this article:** Fisher, D. T. *et al*. Intraoperative intravital microscopy permits the study of human tumour vessels. *Nat. Commun.* 7:10684 doi: 10.1038/ncomms10684 (2016).

## Supplementary Material

Supplementary Information Supplementary Figures 1-5 and Supplementary Tables 1-3

Supplementary Movie 1 Representative video depicting IVM observation of Patient #1 tumour.

Supplementary Movie 2 Representative video depicting IVM observation of Patient #2 tumour.

Supplementary Movie 3 Representative video depicting IVM observation of Patient #10 tumour.

Supplementary Movie 4 Representative video depicting methods for analysis of IVM observation of Patient #3 tumour.

## Figures and Tables

**Table 1:** Human melanoma vascular parameter measurements by IVM and IHC.

Patient	HR	MAP	Tumour vessel measurements by IVM					Tumour vessel measurements by IHC		
			Fields	Visualized vessels	Diameter (μm)	Velocity (μm s^−1^)	% Non-functional	Fields	Visualized vessels	Diameter (μm)
#1	51	59	4	23	35±5.3	253±46	13±13	5	121	12±0.8
#2	58	105	6	44	44±4.5	380±66	44±22	10	92	22±3.4
#3	75	81	4	32	28±3.4	219±73	56±11	5	51	18±2.4
#4	72	81	NA	NA	NA	NA	NA	NA	NA	NA
#5	71	80	4	14	47±4.3	NA	NA	5	133	12±1.0
#6	75	72	5	47	23±3.2	256±52	48±8	5	214	8±0.4
#7	60	79	4	52	24±3.9	234±89	50±19	10	75	16±2.1
#8	85	79	4	37	30±7.1	314±68	74±31	5	111	13±1.8
#9	70	74	3	16	21±5.8	NA	NA	5	14	13±3.0
#10	90	83	4	46	28±6.7	346±92	64±14	10	117	11±0.8

HR, heart rate; MAP, mean arterial pressure; NA, not applicable.

Diameter, Velocity and per cent non-functional are mean±s.e.
