# Supplementary material for: Intraoperative intravital microscopy permits the study of human tumour vessels
Source: Nat Commun. 2016 Feb 17;7:10684. doi: 10.1038/ncomms10684 (PMC4757793; doi:10.1038/ncomms10684)
Supplement: Supplementary Information — Supplementary Figures 1-5 and Supplementary Tables 1-3 [file ncomms10684-s1.pdf]

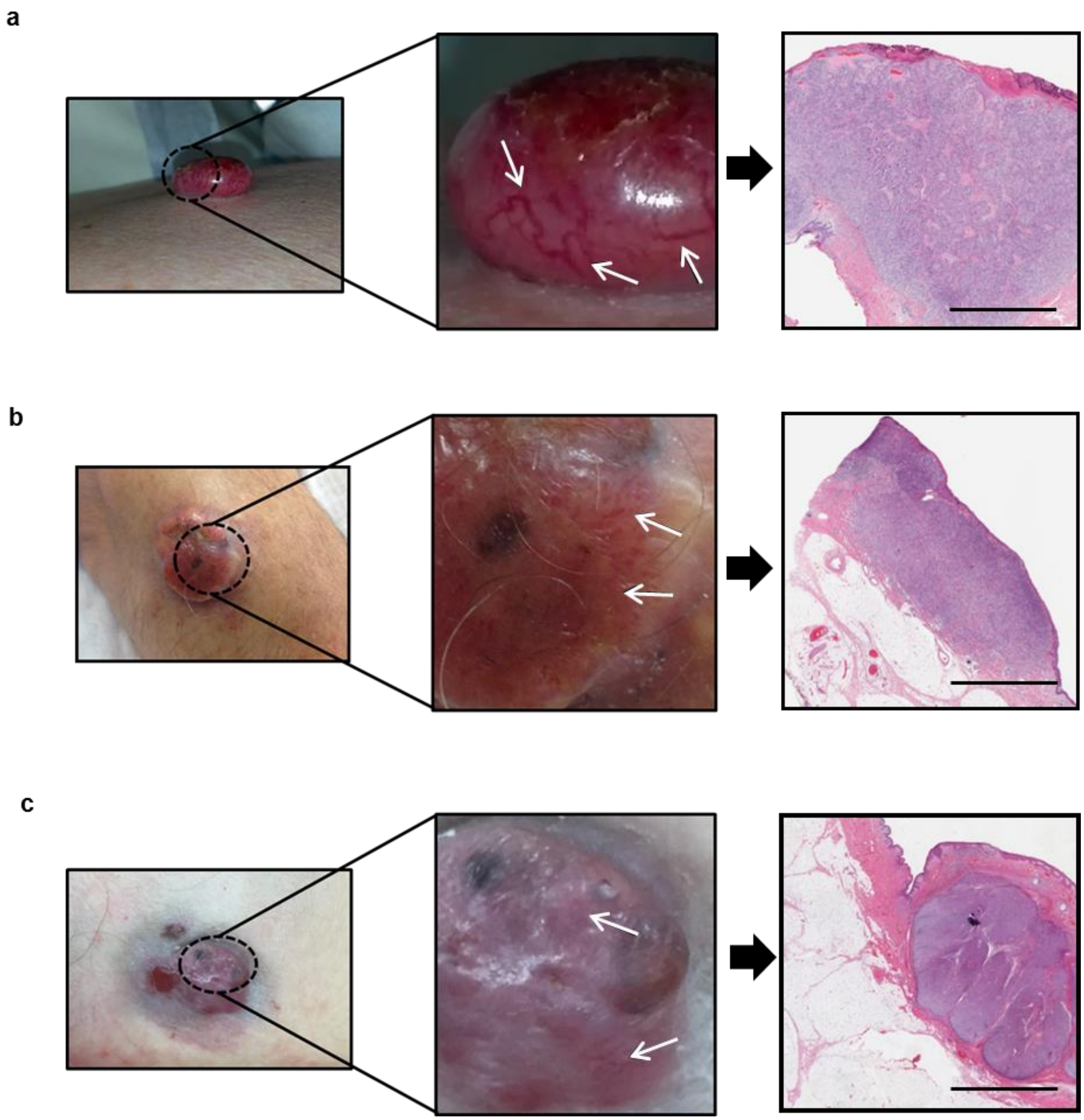

Supplementary Figure 1. Primary melanoma tumors evaluated by transcutaneous IVM. Representative photomicrographs of tumors *in situ* at the time of surgery (Pt #2 A, 7 B, 10 C), inset middle panel demonstrates visible vessels superficially on the tumor surface (white arrows). Right panel depicts hematoxylin and eosin stains of tumors keeping the orientation of observation to facilitate measurements of vessels at the periphery vs. core. Bar is 4 mm.

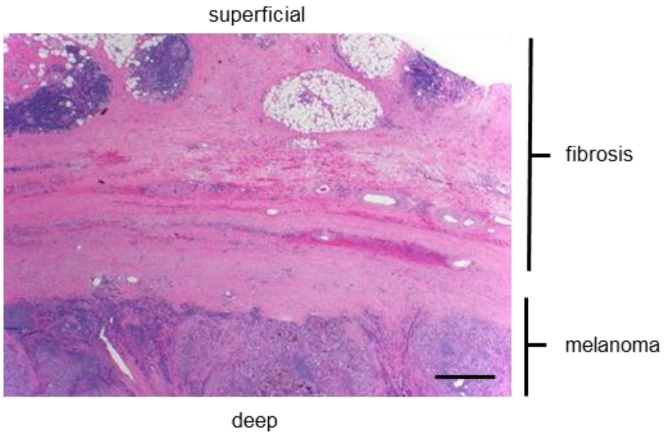

Supplementary Figure 2. Representative histology of Patient #4. Representative H&E stained tissue section shows desmoplastic reaction surrounding the tumor nodule that obscured human IVM observation. Bar is 500 µm.

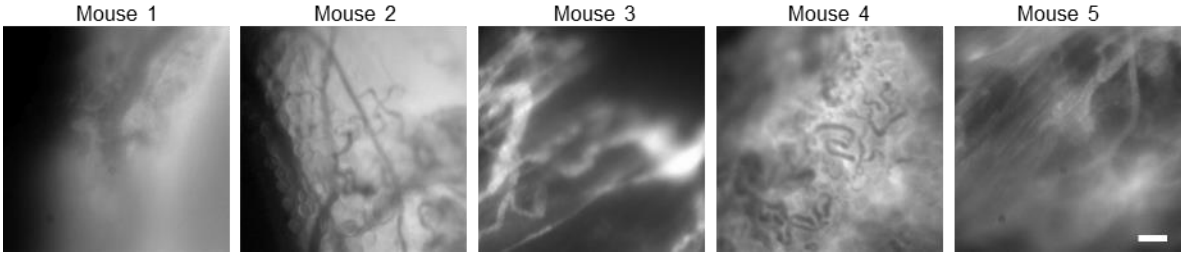

Supplementary Figure 3. Detection of blood flow in murine tumor microvasculature. Representative photomicrographs of tumor microvasculature in mice following fluorescein injection. Bar = 100 µm.

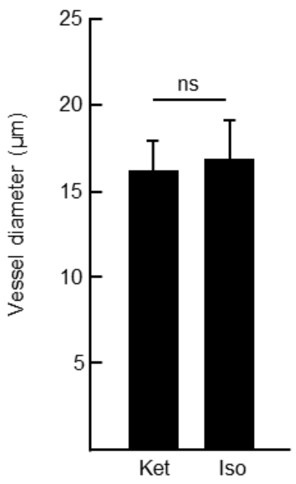

Supplementary Figure 4. Comparison of mouse tumor vessel diameters measured by IVM under ketamine (Ket) and isoflurane (Iso) anesthesia., ns=non-significant. n=4 per group. Data are mean ± s.e.m.

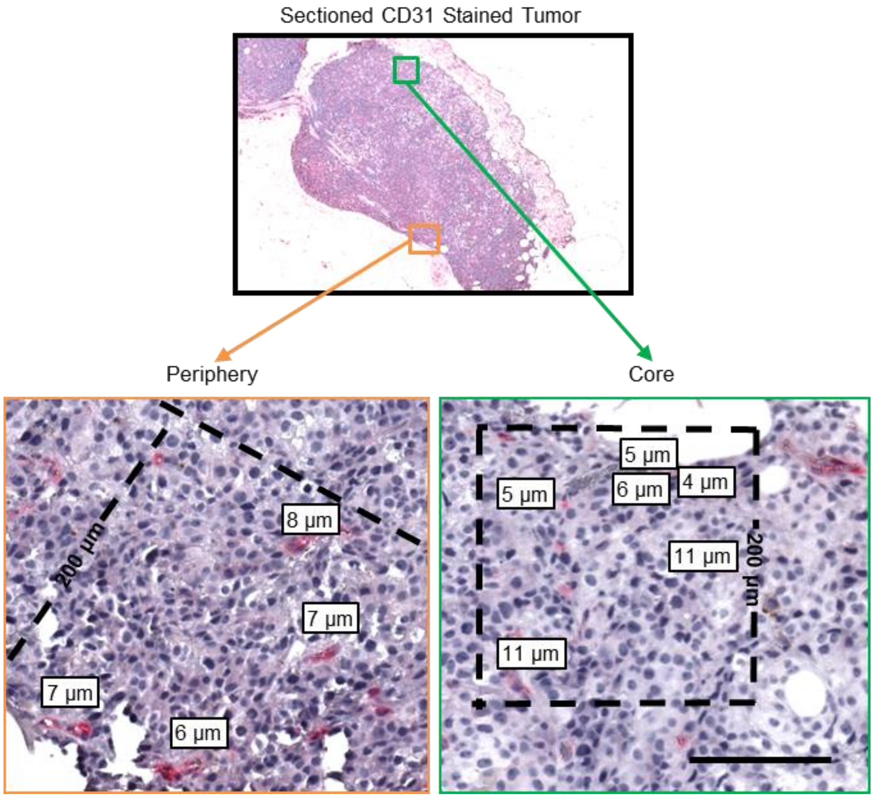

Supplementary Figure 5. Vessel diameters are similar in the periphery and core of murine B16 tumors. Measurements of core (>200 µm from surface) and peripheral (<200 µm from surface) tumor blood vessels was performed on formalin fixed tumor samples stained using hematoxylin and anti-CD31 mAb. Bar is 100 µm.

Supplementary Table 1. Inclusion criteria for human IVM trial.

**Inclusion Criteria**	
	Age ≥ 18 years
	ECOG performance status ≤ 2
	Visible melanoma tumor ≥ 0.5 cm in maximal diameter
	Melanoma tumor that requires an excision in the operating room
	A negative skin-prick test to fluorescein
**Exclusion Criteria**	
	Uncontrolled intercurrent illness
	Melanoma deposit deemed inaccessible to microscopic observation
	Renal dysfunction defined as a GFR < 70
	Prior allergy or reaction to fluorescein including a positive skin-prick test
	Pregnant or nursing females

ECOG = Eastern Cooperative Oncology Group
GFR = Glomerular Filtration Rate

Supplementary. Table 2 - Patient demographics and observation success rates

	Age		BMI				Visualized tumor vessel features		
Patient	(years)	Sex	(kg m^{-2})	Melanoma type	Location	Depth	Diameter	Density	Fluorescein
#1	74	M	29.3	in-transit metastasis	proximal left arm	subcutaneous	+	+	+
#2	49	F	47.1	nodular primary	proximal left arm	dermal/subcutaneous	+	+	+
#3	43	F	37.2	bulky nodal metastasis	left groin	subcutaneous	+	+	+
#4	63	M	32.0	bulky nodal metastasis	left axilla	subfascial	-	-	-
#5	45	F	35.6	in-transit metastasis	right mid back	dermal/subcutaneous	+	+	-
#6	58	F	24.5	bulky nodal metastasis	right groin	subcutaneous	+	+	+
#7	55	M	28.3	nodular primary	right forearm	dermal/subcutaneous	+	+	+
#8	64	F	25.2	bulky nodal metastasis	left groin	subcutaneous	+	+	+
#9	59	M	28.1	bulky nodal metastasis	left axilla	subfascial	+	+	-
#10	73	M	33.2	nodular primary	left groin	dermal/subcutaneous	+	+	+
						Success rates	90%	90%	70%

Supplementary Table 3. Multiple murine tumor models compared to patient tumors

Model System	Tumor vessel measurements by IVM		
	Diameter (µm)	Blood flow velocity ($\mu m\ s^{-1}$)	Wall shear stress ($dyn\ cm^{-2}$)
B16†	13.2±1.4*	242±85	3.22±0.8*
CT26†	14.1±1.5*	244±62	3.05±0.6*
EMT6†	13.5±1.4*	316±76	4.11±1.1*
4T1†	10.8±0.9*	350±93	5.7±0.4*
B16	14.5±1.8*	326±91	4.14±1.1*
Patient	30.5±3.4	286±23	1.69±0.1

Patient and B16 data are reproduced from figure 3b and are provided for comparison

† Performed in the dorsal skin flap window chamber

Diameter, Wall shear stress are mean ± standard error

* = $p < 0.05$ compared to patient vessels
